# Foot care education in patients with diabetes at low risk of complications: a consensus statement

**DOI:** 10.1111/j.1464-5491.2010.03206.x

**Published:** 2011-02

**Authors:** A McInnes, W Jeffcoate, L Vileikyte, F Game, K Lucas, N Higson, L Stuart, A Church, J Scanlan, J Anders

**Affiliations:** School of Health Professions, University of BrightonBrighton; * Foot Ulcer Trials Unit, Nottingham University Hospitals TrustNottingham; † School of Clinical and Laboratory Sciences, University of ManchesterManchester; ‡ Goodwood Court Medical CentreHove; § NHS ManchesterManchester Community Health; ¶ East Cheshire NHS TrustMacclesfield, Cheshire; ** SSL InternationalManchester, UK

**Keywords:** diabetes, education, foot self-care, neuropathy, peripheral vascular disease

## Abstract

**Aims:**

To define and agree a practical educational framework for delivery by all healthcare professionals managing patients with diabetes, particularly those at low risk of developing foot complications.

**Methods:**

A consensus meeting of a multidisciplinary expert panel. Prior to the meeting, relevant clinical papers were disseminated to the panel for review. The consensus was largely based upon the experts’ clinical experience and judgement.

**Results:**

Four main health behaviours were identified for those at low risk of developing foot complications, namely:, control of blood glucose levels; attendance at annual foot screening examination; reporting of any changes in foot health immediately; and the engagement in a simple daily foot care routine.

**Conclusion:**

There is currently little evidence-based literature to support specific foot care practices. Patients with diabetes at low risk of developing complications should be encouraged to undertake a basic foot care regimen to reduce their likelihood of developing complications.

## Introduction

### Overview

There is currently little consistency in the education provided to people with diabetes regarding foot health and foot self-care. Of particular concern are those patients who are considered to be at low risk of developing diabetes-related foot complications. These patients may receive little, if any, information about these complications and how they might be avoided [1–3].

There is little specific evidence-based guidance regarding the content or provision of foot care advice for diabetes patients without established (overt symptoms of) neuropathy or peripheral vascular disease [4–6]. Indeed, only two specific guidelines currently exist for this patient population—those published by the American Diabetes Association and the International Working Group on the Diabetic Foot in 2004 and 2007, respectively [7,8]. The National Institute of Health and Clinical Excellence (NICE) guidance, published in 2004, simply states that ‘healthcare professionals should discuss and agree with patients a management plan that includes appropriate foot care education’ [9].

In some situations, no advice about basic foot health is provided to this group (e.g. the importance of being aware of the possibility of developing diabetes-related changes such as insensate feet at risk for ulceration as a result of peripheral neuropathy) and studies have demonstrated that healthcare professionals are significantly more likely to perform foot examinations and provide foot care education when managing a patient with established foot lesions [10,11]. Whatever the situation, it is widely acknowledged that adequate foot self-care is not undertaken by the majority of patients with diabetes [10,12–14]. To effectively educate diabetes patients, especially those at low risk of complications, on the importance of foot care, it is crucially important that healthcare professionals develop their understanding of the patient perspective. In addition, a necessary adjunct to the patient perspective is the need to recognize those patients who are at a low risk of complications.

### The patient perspective

A number of surveys and studies of patients with diabetes have reported that 23–63% check their feet rarely or not at all [10,12–14]. Other studies have reinforced patients’ lack of understanding that diabetes is a serious illness and the need for preventive measures relating to foot complications, such as changing their shoe-wearing behaviour [15,16].

A recent, as yet unpublished, survey conducted on behalf of SSL International uncovered a number of contradictions in patient perceptions and fears. These included a dichotomy between understanding that a foot care regimen is necessary, but not seeing the tangible benefits of employing such a regimen; or wanting information about their diabetes but not wanting to listen to educational messages.

Further observations during medical consultations [17] and interviews with patients [18] have revealed that patients tend to think that foot problems develop as a consequence of poor blood supply rather than nerve damage. Patients wrongly assume that, if their feet are warm and apparently without symptoms, they are healthy and not in an imminent danger of insensate injury. There is also a belief among patients that foot lesions are accompanied by pain and that gangrene is an inevitable consequence of diabetes, rather than one of the final stages in an essentially controllable pathway that links diabetes and foot ulceration.

People with diabetes may feel let down by healthcare professionals for the lack of adequate foot care advice during the early years following their diagnosis [16]. Ultimately this can impair trust and affect the way that patients interact with healthcare professionals.

### Focus on patients at low risk of foot complications

The National Institute of Health and Clinical Excellence defines low-risk patients as those with normal sensation and palpable pulses [9]. Currently, foot care education is largely targeted at those patients with pre-existing complications, higher HbA_1c_ levels and those who have had diabetes for several years [1]. It seems that foot health education for low-risk patients is not considered to be cost-effective [2,3].However, low-risk patients can develop foot complications relatively quickly in the absence of good glycaemic control and foot self-care practices that facilitate the prompt identification of changes in sensation [19]. Also, it is important to note that most patients with foot complications were categorized as low risk at some point previously. A UK study showed that low-risk patients were less likely to understand the risks related to foot injury, impaired wound healing and the need for daily foot washing than high-risk patients [20].

Therefore, low-risk patients are potentially very vulnerable, highlighting the need for good glycaemic control combined with a basic, daily foot care regimen promptly after diagnosis. Such a regimen would facilitate the early identification of changes indicative of neuropathy.

## Objectives

The objective of this consensus statement is to propose a framework for educating patients with diabetes who are considered to be at a low risk of complications. This framework will focus on the importance of attendance at an annual foot screening appointment, maintaining adequate glycaemic control, self inspecting feet regularly for changes in skin colour, breaks in the skin, swelling or pain and reporting those changes to a healthcare professional. There is a concern that, in the absence of such a consensus among healthcare professionals, uncertainty and avoidance of recommended foot care behaviours may occur among patients with diabetes [5].

To address these concerns this consensus statement will:

- guide healthcare professionals in communicating the importance of basic foot self-care to all people with diabetes, particularly those at low risk of foot complications;
- act as a foundation for further education.

## Method

In June 2009, a multidisciplinary expert panel met to define and agree a practical educational framework for delivery by all healthcare professionals managing patients with diabetes, particularly those at low risk of developing foot complications. The panel comprised diabetologists, podiatrists, a general practitioner, a psychologist and a pharmacist. The general practitioner was able to provide an alternative clinical view to that of the specialist diabetologist and the health psychologist, which provided a patient perspective. Prior to the meeting, a thorough search of the relevant literature was conducted using online databases, namely Science Direct, NHS Evidence, PubMed and the Cochrane Library. The search covered the period from 1995 to 2009 and the search terms used included diabetes, diabetic complications, diabetic foot, foot care, self-care and education. Additional evidence was included from the authors’ knowledge of the literature. The clinical papers this search yielded were reviewed for their relevance to the topic of foot care education in diabetes patients at low risk of complications. Those papers considered to be most relevant were disseminated before the meeting for the experts to review. In addition, the experts were also encouraged to recommend further evidence-based publications. The group meeting was facilitated by the first author and followed a modified nominal group technique [21]. The nominal group technique focuses on a single goal and, in this case, the goal was to establish consensus on the content of the self-care messages that need to be communicated to those patients who are considered to be at low risk of diabetes-related foot complications. These messages represent the basis for the educational framework that will help to inform diabetes patients at low risk of complications on the importance of foot self-care.

## Results

Because of the general paucity of publications regarding this aspect of diabetes management, the consensus was largely based upon the experts’ clinical experience and judgement. With that stated, the overarching aim of this statement is to ensure that patients have an accurate understanding of the key risk factor for foot ulceration, namely, diabetic peripheral neuropathy.

In the first instance, and where resources and time allow, the patient’s level of knowledge about their diabetes, its relationship to potential foot complications and their current foot care regimen should be evaluated, in a non-judgemental manner and using their own language [22]. The abbreviated Patient Interpretation of Neuropathy (PIN) questionnaire and other tools (e.g. the Foot Care Confidence Scale, the Nottingham Assessment of Functional Footcare) may be used to do this [23–27]. This should uncover any misconceptions that the patient may have which can then be discussed and addressed. Using simple and separate models of the nervous and vascular systems can help to initiate discussions about peripheral vascular disease and neuropathy, how they develop and how certain behaviours can influence the progression of these conditions. During the expert discussions, four key educational priorities emerged for low-risk patients:

- attending their annual foot screening appointment;
- maintaining adequate glycaemic control;
- checking their feet regularly;
- reporting any changes in their feet immediately to their healthcare professional.

### Annual foot screening attendance

All patients with diabetes should expect to receive an annual foot screening examination by an appropriately trained healthcare professional and they should feel confident enough to ensure that this takes place. At each step of the foot screening examination, the patient should be told what the healthcare professional is checking or testing for; for example, that testing for sensation is performed to check for nerve damage, rather than for poor circulation [17,27,28]. They should feel confident that the healthcare professional is taking good care of their feet and be informed about the care that they can expect to receive [9].

### Glycaemic control

Numerous clinical studies have demonstrated the positive relationship between reductions in HbA_1c_ and the reduced risk of microvascular complications of diabetes, including neuropathy and foot ulcers [29,30]. This relationship needs to be explained to patients in a language they can understand, together with tackling misconceptions, such as amputation being an inevitable consequence of having diabetes and the link between neuropathy and ulceration. Emphasis should be placed on the fact that many foot complications can be prevented by the patient taking good care of their diabetes; i.e. that they are in control of their blood glucose levels [9]. Ample time should be allowed for the patient to ask questions.

### Checking their own feet

Many patients may not understand the value of checking their feet on a daily basis [23,31]. Demonstrations and visualizations of foot complications and self-care practices may therefore help to engage and empower patients [32] and reinforce that early detection of problems or changes by themselves is key to preventing serious complications [9].

When considering what patients should be looking for when they check their feet, the panel agreed that changes in colour of or breaks in the skin, swelling, pain or numbness are the key features [9].

### Reporting changes in their feet

Perhaps just as importantly as checking their feet, patients also need to be aware that, if they find changes in the colour of the skin, skin breaks, skin swelling, or if they feel pain or numbness in their feet, they should alert their general practitioner or other healthcare professional promptly.

## Discussion

### Education of low-risk patients

The outcomes of education on foot self-care practices among patients with diabetes depend on the type of education provided. Comprehensive educational programmes are associated with substantial increases in the proportion of patients examining their feet daily, conducting other self-care practices and having professional assessments of their feet [1,13,32–35]. There are conflicting data on the effect of education on the incidence of foot complications. Reductions have been noted in some studies [13], while others have reported no effect (35).

Written materials without any input or individualization from a healthcare professional may not be enough to motivate low-risk patients to undertake adequate foot care practices [36–38]. Furthermore, there is no uniform manner in which patients respond and react to healthcare information [39]. The way in which guidance is delivered is therefore essential in order to facilitate the patient’s transition from knowledge acquisition to changing behaviour [39]. Indeed, the National Institute of Health and Clinical Excellence recommends using different educational approaches until the optimal methods are identified [9].

Studies have shown that the accurate interpretation of medical information regarding foot ulcer causes and the nature of foot ulcer risks enhanced preventive foot self-care both directly and indirectly by addressing patient misperceptions [27,28]. Bridging the gap between the patient and practitioner perspectives of foot complications may be the way towards effective foot self-care.

Healthcare professionals must fully engage with patients when discussing foot care, as their level of interest can be perceived by the patient as being directly related to the importance of this topic and the priority that they should assign to it themselves [31]. The motivations to undertake behavioural change, such as setting aside time every day to check their feet, must also be considered during these discussions [32].

Diabetes is a silent disease until the onset of overt complications, so there is no symptom-driven motivation to change behaviour, especially in the early stages of the condition [16]. Instead, behaviour may be altered by perceptions regarding the impact of diabetes on everyday life, patients’ ability to exert control over their condition and the effectiveness of preventive strategies, i.e. their health beliefs [16,36,39]. In view of these considerations, targeted education should therefore begin with an evaluation of the patient’s health beliefs and their desire to engage in performing foot self-care.

Any educational programme must also allow for a certain amount of individualization to allow for different patients with different personal circumstances. For example, the needs and educational requirements of a young patient with Type 1 diabetes and an older patient with Type 2 diabetes will differ considerably. Similarly, we must make allowances for patients who may require assistance with checking their feet as a result of visual impairment or mobility problems [20,40]. In addition, the provision of relevant information may be insufficient to influence the behaviour of patients who lack confidence in performing self care practices [39]. Indeed, it has been documented that greater self-efficacy, i.e. confidence in performing health-related behaviours, is associated with a greater likelihood of performing foot self-care practices [14].

The National Institute of Health and Clinical Excellence currently recommends that low-risk patients should receive a specific management plan that includes foot care education, in order to improve their knowledge, minimize accidental injury and encourage beneficial self-care [9]. Many low-risk patients may not see a specialist healthcare professional on a regular basis, so it is important that all healthcare professionals seeing such patients communicate the same messages in a consistent way.

### Timing of education

The potential consequences of poor foot care in diabetes are grave; therefore, the importance of foot health must be communicated at an early stage. However, healthcare professionals need to be cognisant of the huge amount of information a patient is expected to assimilate when they are diagnosed with diabetes and the risk of information overload [31].

Foot care education is an integral part of the diabetes information package, but it needs to be delivered in a way that is sympathetic to how the patient in question deals with their diagnosis and how able they are to absorb new information.

At the very least, a patient should receive foot care education and guidance during their annual foot screening examination. Several studies have shown that an intensive one-off education programme or session following diagnosis of diabetes may achieve improvements in self-care in the short-term, but that these are not maintained over a longer period [16,32,41]. Maintaining these improvements in the long-term is dependent on reinforcing messages and providing opportunities for questions during each visit.

## Conclusions

There is currently little evidence-based literature to support certain foot care practices. However, this consensus meeting allowed the identification of a number of key elements that need to be communicated in any educational initiative. These elements relate to the management of diabetes as a whole, the timing of foot health assessments by healthcare professionals, reporting any changes in foot health to a healthcare professional and the importance of self-care practices.

These key educational elements for diabetes patients at low risk of complications are captured with the mnemonic CARE:

- Control: control blood glucose levels (in accordance with recommendations from your healthcare professional).
- Annual: attend your annual foot screening examination with your healthcare professional.
- Report: report any changes in your feet immediately to your healthcare professional.
- Engage: engage in a simple daily foot care routine by washing and drying between your toes, moisturizing and checking for abnormalities.

Encouraging patients with diabetes at low risk of foot complications to undertake a basic foot care regimen is of critical importance. Basic foot care is simple, quick and empowers the patient in managing their diabetes more proactively, thus reducing the likelihood of complications later on.

Educational initiatives, based on the CARE framework above, should be tailored to the individual and take into account their health beliefs, motivation to change and personal circumstances. The importance of reinforcing the principles of the CARE framework on a regular basis cannot be overstated.
